# Notes

**Published:** 1899-09-23

**Authors:** 


### NOTES.

The Glasgow Corporation propose to give a salary of £350 per annum to the bacteriologist to the Corporation departments, whom they are about to appoint.

The Jenner Institute of Preventive Medicine has obtained the sanction of the High Court (Chancery Division) to the alteration of their memorandum of association, which was necessary in order to enable them to benefit by the gift of £250,000 made by Lord Iveagh.

Once more the London hospitals are offering the full complement of accommodation to sufferers after the summer, which lessens the tax upon them. King's College Hospital has been much improved during the time it has been closed. The wards have been refloored, and the electric light has been introduced. The Westminster Hospital reopens with a new out-patients' department.

Considerable anxiety has recently been caused at Aldershot by the occurrence of a series of cases of gastro-enteritis among the troops who have recently returned from the evolutions on Salisbury Plain. Three men have died of enteritis, and there have been from 30 to 40 men in hospital suffering from a similar condition. The intestines from one of the fatal cases have been sent to London for more complete examination.

At a recent meeting of the Dublin Public Health Committee it was stated that the death-rate from all causes in the city was at the present time 42·4 per 1,000 living; and Sir Charles Cameron reported that there was a serious epidemic of measles in Dublin and its suburbs, which would probably continue for many weeks. Numerous cases of typhoid fever also had occurred in the Constabulary Depôt. This outbreak began to show itself about a month ago, since when cases had continued to occur.

